# Tailoring Treatment in Complex Regional Pain Syndrome: A Comparative Study of Therapeutic Approaches in Complex Rehabilitation

**DOI:** 10.3390/ph18081114

**Published:** 2025-07-25

**Authors:** Iana Andreieva, Beata Tarnacka, Adam Zalewski, Justyna Wiśniowska

**Affiliations:** 1 Department of Rehabilitation Medicine, Faculty of Medicine, Warsaw Medical University, Spartańska 1, 02-637 Warsaw, Poland; beata.tarnacka@wum.edu.pl; 2 Department of Rehabilitation, Eleonora Reicher National Institute of Geriatrics, Rheumatology and Rehabilitation, Spartańska 1, 02-637 Warsaw, Poland; adam@drzalewski.pl (A.Z.); justyna.wisniowska@spartanska.pl (J.W.)

**Keywords:** complex regional pain syndrome (CRPS), analgesic treatment, rehabilitation, pain management, DASH, LEFS, neuropathic pain

## Abstract

Complex regional pain syndrome (CRPS) is a disabling pain condition, which is distinct from other pain syndromes by the presence of autonomic dysfunction and regional inflammatory changes. **Objectives:** To explore the impact of pharmacological treatment strategies, specifically scheduled, on-demand dosing regimens versus lack of medical treatment, on pain-related and functional outcomes in rehabilitation for individuals with CRPS. **Methods:** A total of 32 participants with CRPS were assigned to three treatment groups depending on analgesic treatment during the course of complex rehabilitation. Pre- and post-rehabilitation assessments were conducted using validated measures, including the Numerical Rating Scale (NRS) for pain, the Short-Form McGill Pain Questionnaire (SF-MPQ), PainDETECT, the Disabilities of the Arm, Shoulder, and Hand (DASH), and the Lower Extremity Functional Scale (LEFS). **Results:** Significant improvements in pain and upper limb function (DASH scores) were observed across all groups (*p* < 0.05). No statistically significant changes were found in lower limb function (LEFS). Between-group comparisons revealed significant differences in post-treatment pain scores (SFMPQ-B), particularly between groups with a constant treatment regimen and those without treatment. **Conclusions:** There were no statistically significant changes compared to different treatment regimen groups. The constant treatment group showed slightly better average improvements in pain and disability compared to other groups. Statistically significant improvements in all CRPS patients were observed in pain-related and functional measures.

## 1. Introduction

Complex regional pain syndrome (CRPS) is a disabling pain condition, which is distinct from other pain syndromes by the presence of autonomic dysfunction and regional inflammatory changes, with a prevalence of approximately 5.4–26.2 per 100,000 people [1,2]. Significant pain, sensory changes, temperature disturbances, discoloration, swelling, abnormal sweating, and trophic changes to nails, hair, and skin characterize it. CRPS is a disorder with predominant neuropathic pain, which is characterized by ongoing pain, mainly disproportionate to the degree of tissue injury. The pain often persists beyond the usual expected time for tissue healing [3,4,5].

Complex regional pain syndrome (CRPS) pathophysiology is complex and not fully understood, but it is believed to involve a combination of factors including nervous system sensitization, autonomic dysfunction, and inflammatory changes [6]. 

Patients with CRPS generally require support to manage pain, functional limitations, and the broader effects of the condition [6].

There is a recognized lack of well-established treatment guidelines specifically for pharmacotherapy in CRPS. Over the last few decades, several pharmacological treatments (such as bisphosphonates, corticosteroids, ketamine, scavengers/MgSO_4_, NSAIDs/selective inhibitors of cyclooxygenase-2, or anti-epileptics) have been proposed for CRPS-I in adults, but up-to-date information regarding the exact efficacy and safety of the various proposed therapeutic agents for this disease is scarce [7]. Many recommendations are formulated based on smaller studies, expert consensus, and clinical practice [8,9]. However, a general consensus emphasizes a multidisciplinary approach that integrates pharmacotherapy with rehabilitation strategies [10].

Rehabilitation plays a vital role in the management of CRPS, with physical and occupational therapy recognized as fundamental components of early intervention [11]. These therapies encompass a range of techniques, including gentle exercises to improve mobility, desensitization protocols to reduce sensitivity, mirror therapy to address pain and function through visual feedback, graded motor imagery to retrain the brain, and tactile discrimination training to enhance sensory processing [12].

Pharmacotherapy serves as a crucial adjunct to rehabilitation in CRPS management. Pain control achieved through medication is often necessary to enable patients to actively participate in the demanding exercises and activities that form the core of rehabilitation programs. The primary aim of using pain medication in this context is to alleviate pain and associated symptoms to a degree that allows for meaningful engagement in rehabilitation [7]. The close coordination between pain control interventions, such as the use of medication, and physical or occupational therapy is essential in disrupting the cycle of pain and disability that characterizes CRPS [13,14].

Different analgesic regimes are employed in CRPS rehabilitation, including scheduled (constant) dosing and on-demand (pro re nata or PRN) dosing.

The choice of medication regime in CRPS rehabilitation is influenced by several factors, including the severity and consistency of the patient’s pain, their ability to understand and manage on-demand medication, the type of medication being used (whether it is short-acting or long-acting), the specific goals of therapy (e.g., achieving consistent baseline pain control versus managing breakthrough pain), the potential for side effects, and the need for stable drug levels [15,16]. Scheduled dosing may lead to better overall pain control and facilitate more consistent participation in therapy. It is often preferred when pain is continuous or present for a significant portion of the day. On-demand medication can empower patients to take control of their pain management and address acute increases in pain. Often, the optimal approach involves a combination of scheduled and on-demand medications to provide both baseline pain control and the flexibility to manage acute pain exacerbations. The severity of the patient’s pain, particularly if it significantly interferes with their ability to perform daily activities and actively participate in rehabilitation exercises, is a primary consideration [17,18].

There is a noticeable absence of comprehensive articles specifically addressing the comparative effectiveness of different analgesic regimens. While many studies focus on individual drugs or general pain management strategies, few systematically evaluate and contrast the outcomes of various analgesic protocols across different clinical settings [19,20]. This gap limits the ability of clinicians to make fully informed, evidence-based decisions tailored to specific patient needs. Emerging research suggests that scheduled dosing of neuropathic agents (e.g., pregabalin or duloxetine) may enhance adherence to therapy programs by providing more stable analgesia, although individualized approaches remain necessary [19]. More recent studies, such as randomized controlled trials comparing gabapentin versus amitriptyline or ketamine infusions versus placebo, have demonstrated short-term pain relief benefits, but their impact on long-term rehabilitation engagement and functional improvement is less clear [20]. There was no assessment of different treatment regimes, only the effectiveness of the therapy. These findings underscore the need for further investigation into how different dosing strategies directly influence rehabilitation outcomes in CRPS, beyond pain relief alone.

This study aims to explore the impact of pharmacological treatment strategies and specifically scheduled, on-demand dosing regimens versus lack of medical treatment on pain-related and functional outcomes in rehabilitation for individuals with CRPS.

## 2. Results

### 2.1. General Assessment

The first step in the analysis was to compare the general CRPS group results obtained on the pre-test and post-test for all outcome measures. All patients with CRPS showed some level of improvement across multiple outcome measures from baseline to Week 4. The most consistent improvements were observed in the primary outcome (pain reduction NRS, PDQ, SFMPQ) and upper limb function (DASH). There was a significant improvement in neuropathic pain, as measured by the PDQ scale (*p* = 0.00079). Sensory pain also showed notable positive changes on both SFMPQ-A and SFMPQ-B scales, with especially strong results observed on the SFMPQ-B. Additionally, a significant reduction in overall pain intensity was recorded on the NRS (W = 71,0, *p* < 0.001). However, no significant changes were found in leg functionality, as indicated by the LEFS scores (W = 261, 0, *p* = 0.532) (Table 1).

Statistical analysis revealed no significant differences between males and females across any of the outcome measures. However, descriptive trends suggest that females tended to exhibit greater improvements in pain and functional outcomes, particularly on the SF-MPQ, PDQ, and DASH scales. While these observations are preliminary and not statistically conclusive, they may reflect gender-related differences in pain perception, coping strategies, or response to multimodal rehabilitation, which warrant further investigation in larger, controlled studies.

No meaningful differences were observed across CRPS types for most outcome measures. However, scores on the LEFS showed a possible trend toward variation between groups (*p* = 0.074), but the unequal number of participants across groups limits direct statistical comparisons.

### 2.2. Comparison Analysis of Treatment Plan

Significant differences were found for secondary outcomes in comparison analysis of the treatment regime between three treatment groups: SFMPQBW4 (Sensory pain component at Week 4) and LEFSW4. Most other metrics, including DASH, NRS, CSIA, and PDQ scores, did not show significant differences across groups (Table 2).

Participants receiving constant treatment (Group 1) demonstrated slightly greater average improvements in both pain and disability measures compared to Group 2 and Group 3. Specifically, Group 1 showed the largest reduction in DASH scores (−14.30), indicating improved upper limb function, and a consistent decrease in pain intensity across NRS (−1.70), CSIA (−1.73), SFMPQB (−29.67), and PDQ (−1.55). Although Groups 2 and 3 also showed improvements, the magnitude of change in these outcomes was generally smaller or more variable. Group 1 maintained a modest gain in lower extremity function (LEFS: +1.14), while Group 2 experienced a decline (−1.78), and Group 3 had the greatest LEFS increase (+4.12), albeit with more variability in other domains.

**Figure 1:**
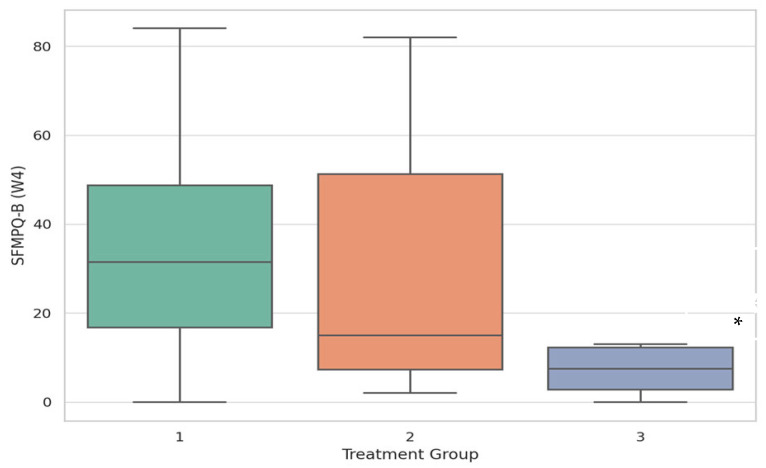
Results of post hoc analysis for SFMPQ-B scores in treatment groups (between-group comparison analysis). Boxplots depict the distribution of SFMPQ-B scores at Week 4 across three treatment groups. Post hoc Mann–Whitney U tests were used for pairwise comparisons. Group 1 and Group 3 differ significantly (*p* < 0.05). *—significant differences (*p* < 0.05). A significant difference was observed between Group 1 and Group 3 (*p* = 0.027), indicating that Group 3 had a significantly lower sensory pain score after 4 weeks. No statistically significant differences were found at the 0.05 level; however, there was a trend suggesting Group 3 had better functional improvement (Group 2 vs. Group 3: *p* = 0.055; Group 1 vs. Group 3: *p* = 0.084).

To determine which groups differed significantly for the key metrics, post hoc pairwise comparisons were performed. Group 1 and Group 3 differ significantly (Figure 1).

### 2.3. Comparison Subanalysis of Anticonvulsants Effectiveness

We did a comparison subanalysis of functional improvement in patients with treatment with anticonvulsants (11 patients) in comparison with patients with CRPS without anticonvulsants treatment (21 patients). The subgroup with pregabalin/gabapentin in complex pharmacotherapy differed in baseline pain scores (NRS), with this group likely starting with higher pain.

**Figure 2:**
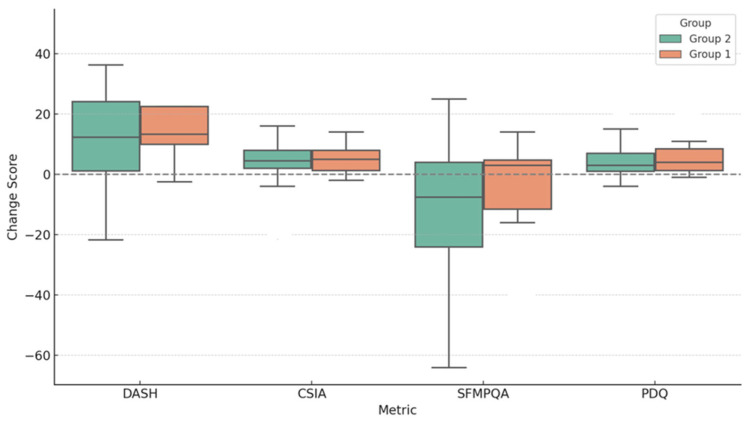
Changes in parameters in subgroups with and without anticonvulsants (pregabalin/gabapentin). Boxplots show change scores (Week 4—Baseline) for four outcome measures (DASH, CSIA, SFMPQA, and PDQ), stratified by Group 1 (with anticonvulsants) and Group 2 (without anticonvulsants). Mann–Whitney U tests were used for group comparisons. Positive values indicate improvement, though not significant.

There is a suggestion of a difference in affective pain components at baseline. All measures—including post-treatment values like NRSW4, DASHW4, and LEFSW4—did not differ significantly between the two subgroups. However, the gabapentin subgroup showed numerically better improvements in pain and function (especially NRS, DASH, LEFS, and PDQ), though not significant—possibly due to a small sample size, which needs further investigation in larger, controlled studies (Figure 2).

## 3. Discussion

This study aimed to assess the influence of different approaches to analgetic treatment on the effectiveness of complex rehabilitation in patients with CRPS by comparing pre-test and post-test outcomes across various measures.

Thirty-two participants completed the project. The study was conducted in three parallel groups with different regimes of analgesic treatment. The evaluation of pain and functional assessment were realized by validated instruments (NRS, SF-MPQ, CSI, PDI). These tools offer a multidimensional approach to pain assessment, facilitating a deeper understanding of pain mechanisms and enabling tailored treatment strategies. They are also valuable for evaluating clinical outcomes and tracking changes in the patient’s condition over time.

The results of complex rehabilitation in all patients with CRPS demonstrated positive changes in several important areas, with notable improvements in pain reduction and upper limb function observed across all groups. Notably, neuropathic and sensory pain levels decreased significantly, and upper limb function, as measured by the DASH scale, showed consistent positive change. However, despite these improvements, leg functionality, as assessed by the LEFS score, did not exhibit any significant changes.

There were no significant statistical differences in pain and functional outcomes between different types of CRPS. At the same time, LEFS demonstrated a non-significant tendency in functional improvement, indicating potential variability in lower extremity function between CRPS types. Post hoc analyses were not applicable in this case due to insufficient subgroup CRPS type sizes—the second group contained only two participants (*n* = 2), with limiting statistical power. CRPS I and II currently do not differ in treatment choice and there is a lack of evidence on whether treatment responses to intervention differ based on subtype of the disease.

The main aim of our study was to assess the influence of different approaches to pharmacological treatment on the results of complex rehabilitation. The constant treatment group (Group 1) showed better average improvements in pain and disability compared to Groups 2 and 3. However, due to high variability and small sample sizes, these differences did not reach statistical significance. In post hoc analysis, a significant difference was observed between Group 1 and Group 3, indicating that Group 3 had a significantly lower sensory pain score after 4 weeks. At the same time, no statistically significant differences were found for other parameters; however, there was a trend suggesting Group 3 had better functional improvement. This may have been related to the initially better functional parameter scores in Group 3, as well as lower baseline pain scores. Group heterogeneity could have played a role. Group 3 included individuals with varying CRPS subtypes, symptom durations, and levels of baseline functional impairment. At the same time, the medication-free group may have been more responsive to non-pharmacologic interventions such as desensitization, biofeedback, or mirror therapy due to lower baseline levels of central sensitization or greater neuroplastic adaptability. This inconsistency underscores the importance of interpreting average trends with caution, particularly when group sizes are small and baseline characteristics differ. Importantly, the study design did not control for such baseline disparities, limiting the causal interpretability of between-group comparisons.

According to the literature reviews, scheduled dosing, also known as around-the-clock dosing, aims to maintain a consistent level of pain relief by administering medication at regular intervals [21,22,23]. This approach may lead to better overall pain control and facilitate more consistent participation in therapy. It is often preferred when pain is continuous or present for a significant portion of the day [24]. Some studies suggest that scheduled dosing is beneficial for chronic pain management [25,26]. One study indicates that the effectiveness of acetaminophen improves with scheduled administration, but this study was conducted with patients with acute pain [27]. Unfortunately, specific evidence comparing scheduled versus on-demand dosing in CRPS rehabilitation within the provided material is limited. Scheduled dosing may be particularly beneficial in providing a baseline level of pain control, making it easier for patients to engage in consistent rehabilitation efforts. These results suggest that consistent treatment may contribute to more stable and slightly more favorable outcomes in CRPS management.

On-demand medication can empower patients to take control of their pain management and address acute increases in pain, but it may not be sufficient for providing the consistent baseline pain relief often required for effective rehabilitation.

The selection of a medication regimen during CRPS rehabilitation depends on multiple factors and must be individualized, taking into account the intensity and variability of the patient’s pain, their ability to appropriately use on-demand medications, the characteristics of the drug, and the primary objectives of treatment and potential side effects.

Several studies support that gabapentin and amitriptyline are relatively safe and effective in improving pain to various degrees and reducing the sensory deficit in the affected limb in CRPS I [28,29,30]. For this reason, we decided to do an additional subanalysis of the influence of anticonvulsant treatment. Anticonvulsants, including gabapentin and pregabalin, are widely used in the treatment of neuropathic pain associated with CRPS. There is moderate evidence suggesting that gabapentin can improve pain symptoms such as hyperesthesia and allodynia [31,32]. A literature review noted that while some randomized controlled trials found gabapentin to result in significant pain improvement, overall evidence remains insufficient to conclusively support its use for CRPS-related pain [33,34]. According to results of our study, there is a suggestion of difference in affective pain components at baseline. Although the gabapentin group showed better outcomes in pain and functional domains, none of these differences reached statistical significance. Given the limited sample size and heterogeneity of treatments, these findings are best considered preliminary and hypothesis-generating. They should not be used to draw conclusions about efficacy in CRPS management without larger, controlled trials.

Most rehabilitation treatments and efforts primarily aim to control patients’ pain, but satisfaction is low and rehabilitation patients with CRPS demand not only analgetic but multidimensional approaches. At the same time, it is not possible to provide successful rehabilitation and treatment for these patients without sufficient pain control. For this reason, further research on treatment standard optimization is needed.

This study has some weaknesses. One of the limitations was the small number of participants. Since CRPS is a rare disease and in combination with diagnostic difficulties, we could not recruit enough patients. With only 32 participants and substantial imbalance between groups (e.g., one group with only two patients), the study was underpowered for detecting moderate treatment effects or conducting reliable subgroup analyses. This study was not registered in a clinical trial database. A further limitation would be the potential for the study not to be fully blinded and lack of randomization, as well as lack of follow-up period which may capture the long-term effects of rehabilitation interventions on CRPS symptoms and patient functioning. Treatment regimens varied considerably in medication type and complexity (e.g., NSAIDs, neuropathic agents, opioids). This heterogeneity is acknowledged as a potential source of confounding and a limitation in internal validity.

One of the limitations was the absence of baseline standardization. Baseline pain and function levels varied notably between groups, which may confound treatment effects. No a priori power calculation was performed due to the exploratory nature of the study. This could be a methodological limitation that may affect the interpretability and generalizability of the findings.

Future research should focus on conducting more large-scale, randomized controlled trials to better establish the efficacy of various pharmacologic agents and regimes in CRPS rehabilitation. Further investigation is needed to determine the optimal ways to integrate pharmacotherapy with different rehabilitation modalities. The development of more specific and evidence-based guidelines for pain medication use in this context is also crucial [21].

Further research with larger, more balanced cohorts and longer follow-up periods is warranted to validate these results and explore differential treatment responses.

## 4. Materials and Methods

### 4.1. Study Design

This was a prospective single-center observational comparative study with three parallel treatment groups. The study was conducted between 10 October 2021, and 1 March 2025, in a clinical rehabilitation setting. Participants were admitted either to an inpatient rehabilitation department or a day rehabilitation ward based on medical referral. This study was not registered in a clinical trial database. It is a pilot study.

#### Eligibility Criteria

The study inclusion criteria assumed clinical diagnosis of CRPS in accordance with Budapest Criteria [35,36,37], age above 18 years, no changes in analgesic therapy for at least 2 weeks before inclusion in the study, and signed informed consent. The exclusion criteria were a non-CRPS diagnosis, age below 18 years, less than 3 months of CRPS history (acute phase of CRPS), or patients refusing to consent to their participation in the study.

A total of 41 participants were initially recruited. Of these, 32 participants completed the study. Dropouts (*n* = 9): suspected stroke (*n* = 1), withdrawal of informed consent (*n* = 2), withdrawal from psychological intervention (*n* = 3), suspected infection (*n* = 1), failure to meet CRPS criteria (*n* = 2).

Thirty-two participants (25 females and 7 males) completed the project. The mean age of all the participants was 54.34 years (range: 28–75 years) (SD = 13.2). The mean duration of illness was −6,3 months (SD = 1.2).

Twenty-eight of the participants met the criteria for type I CRPS; 2 had a diagnosis of type II CRPS and 2 were diagnosed with RSF CRPS.

Etiology: fracture (65%), trauma (30%), post-surgical with immobilization (30%), conservative treatment with immobilization (60%)

Most cases involved the upper extremity (*n* = 25; 78.1%), while 7 patients (21.9%) had CRPS affecting the lower extremity.

CRPS was diagnosed between 3 and 18 months after the inciting event (trauma). The recruitment of patients to the program came most often within a year after the CRPS diagnosis.

### 4.2. Assessment

Before (pre-test) and after (post-test) rehabilitation, all participants had two medical, psychological, and physiotherapeutic assessments. Due to eligibility criteria, all of the participants before being admitted to the program had to undergo a thorough medical examination. The medical assessment included various scales and questionnaires that examined the affected limb condition and neuropathic component of pain. The physiotherapist examination included especially a possible range of motion and muscle strength.

To evaluate pain, function, and the potential presence of neuropathic or centrally mediated pain, the following validated instruments were administered both before and after the 4-week rehabilitation program:

- Numerical Rating Scale (NRS);
- Short-Form McGill Pain Questionnaire (SF-MPQ);
- PainDETECT Questionnaire (PDQ);
- Central Sensitization Inventory (CSI).

All questionnaires were self-administered.

Pain was measured using the Numeric Rating Scale (NRS). The NRS is a self-reported tool that measures the average pain intensity over the previous 24 h with possible scores ranging from 0 (no pain) to 10 (worst possible pain). Patients reported pain intensity scores every day.

Two widely recognized patient-reported outcome measures used for this purpose are the Lower Extremity Functional Scale (LEFS) and the Disabilities of the Arm, Shoulder and Hand (DASH) questionnaire.

The MPQ also includes a Present Pain Intensity (PPI) index and a Visual Analogue Scale (VAS) for overall pain severity.

Results of pain assessment with NRS were considered as primary outcomes. Evaluation of function and the potential presence of neuropathic or centrally mediated pain were considered as secondary endpoints. All primary and secondary outcomes were measured two times: at baseline (pre-test) and after 4 weeks (post-test).

### 4.3. Rehabilitation Program

The study included a 4-week Multimodal Rehabilitation Program with physical therapy, physiotherapeutic, and psychological interventions dedicated to CRPS participants.

The rehabilitation program included (1) individual physiotherapy (15 min), (2) individual manual hand/foot therapy (20 min) with desensitization, (3) Transcutaneous Electrical Nerve Stimulation (TENS, 20 min), (4) biofeedback (15 min), and (5) psychological interventions (45 min) as pain psychoeducation and relaxation.

Individual physiotherapy (kinesitherapy) was aimed at reducing pain and improving the range of mobility of the affected limb. The main techniques involved fascial therapy, Myofascial Release, joint mobilization, passive and action exercises, and action exercises in water. Individual manual hand/foot therapy was carried out using hand/foot devices aimed to improve manual dexterity in combination with desensitization. Physical therapy included hydrotherapy and TENS, using thermal and mechanical stimulation of the affected limb. Hydrotherapy was used to decrease pain intensity, reduce swelling, and improve blood circulation [38]. TENS has an analgesic effect, based on Melzack and Wall’s theory of stimulation of nerve fibers [39].

During the physical therapy interventions, TENS machine (Technomex Multitronic MT-3 produced by Technomex Sp. z o.o., Gliwice, Poland) and hydrotherapy for the upper and lower limbs (Technomex, model 1114T and 1117, produced by Technomex Sp. z o.o., Gliwice, Poland) were used.

The Neuroforma device (Neuroforma MobileBox equipped with a Kinetic video camera, a 28-inch screen, and computer program, produced by Titanis Sp. z o.o., Warsaw, Poland) works on the basis of motion capture using a webcam and enables the analysis and correction of a virtual image (augmented reality). The participants were seated in front of the screen (1.5 m away). The following apparatus was used during the psychological interventions: iPad (8th Generation, 14.8 iPadOS software) with Noigroup’s Hand Recognise and Foot Recognise Apps installed to train the first two stages of GMI (left/right discrimination, explicit motor imagery), and a Medilab Therapy Mirror Box (size of the box open: 35 × 24 × 24 cm, size of the acrylic mirror: 31.5 × 21.5 × 1.5 cm) for the third stage of GMI (mirror therapy) with both upper- and lower-limb-affected patients and the mirror therapy module.

Rehabilitation sessions were conducted 5 days per week, and the rehabilitation intensity was identical across all groups. Interventions were delivered by licensed physical therapists and clinical psychologists.

The 4-week Multimodal Rehabilitation Program was the same for the stationary rehabilitation department and the daily rehabilitation department.

### 4.4. Treatment

The study compared outcomes across groups stratified by pre-existing analgesic regimens. No randomization or blinding was performed. Participants were allocated based on their ongoing pharmacological pain management strategies at least 2 weeks before and during the rehabilitation program, which were initiated independently by their primary care physicians and were not altered by the research team. The treatment plans were established at least one month before the start of the rehabilitation program and remained unchanged for a minimum of two weeks before enrollment. Patients were not randomized into treatment groups. Treatment regimens varied considerably in medication type and complexity (e.g., NSAIDs, neuropathic agents, opioids) and were the same for the stationary rehabilitation department and the daily rehabilitation department. All pharmacological treatments were administered orally, and no injectable or transdermal routes were used. Medication regimens reflected standard clinical practice.

The treatment groups were as follows:

1. Constant Analgesic Therapy Group (Group 1)

Patients received continuous pharmacological pain management on a scheduled basis, regardless of pain fluctuations.

2. On-Demand Analgesic Therapy Group (Group 2)

These patients took analgesics only when needed, based on their pain experience. This approach was patient-controlled.

3. No Pharmacological Pain Treatment Group (Group 3)

Patients in this group were not using any regular pharmacologic pain treatments during the study period and had not received pain medications for at least two weeks prior to rehabilitation. Any pain management was addressed through non-pharmacologic measures.

The clinical characteristics of the included patients are shown in Table 3.

Pharmacological therapy varied among participants, with the most frequently used medications represented in Table 4.

In Group 2, patients were prescribed medications on a pro re nata (PRN) basis. The average number of medications per patient was 1.5, with the most common being NSAIDs and tramadol. The medications were taken as needed, averaging 2–4 days per week. Doses varied, but standard PRN doses included the following:

Ibuprofen 400 mg

Tramadol 50 mg

Paracetamol 500–1000 mg

Twelve participants (37.5%) were not receiving any pain medication at the time of enrollment. Treatment combinations ranged from monotherapy to complex polypharmacy regimens.

The research protocol was approved by the Ethics Committee. All procedures involving human participants were conducted in strict accordance with the ethical standards outlined in the Declaration of Helsinki, as revised in 2013. Informed consent was obtained from all participants, and confidentiality of personal data was maintained throughout the study.

### 4.5. Outcome Measures

Assessments were conducted at two time points:

T0 (Baseline): Prior to program initiation

T1 (Post-Test): At the end of the 4-week rehabilitation

Primary Outcome

Pain intensity: Assessed using the Numerical Rating Scale (NRS), a self-reported measure (0 = no pain; 10 = worst possible pain), recorded daily.

Secondary Outcomes

Short-Form McGill Pain Questionnaire (SF-MPQ): Sensory and affective dimensions, Present Pain Intensity (PPI), and Visual Analogue Scale (VAS)

PainDETECT Questionnaire (PDQ): Assessment of neuropathic pain component

Central Sensitization Inventory (CSI)

Functional Capacity:

Lower Extremity Functional Scale (LEFS) for lower-limb CRPS

Disabilities of the Arm, Shoulder, and Hand (DASH) for upper-limb CRPS

All questionnaires were self-administered, validated, and applied consistently at T0 and T1.

### 4.6. Statistical Analysis

No a priori power calculation was performed due to the exploratory nature of the study. We acknowledge this as a methodological limitation that may affect the interpretability and generalizability of the findings.

All analyses were carried out using IBM SPSS Statistics 28 (produced by IBM, Armonk, NY, USA).

In the beginning, the differences among demographic and clinical variables via one-way repeated measures ANOVA with post hoc Mann–Whitney U test were calculated. 

The normality of data distribution was assessed using the Shapiro–Wilk test. The results indicated non-normal distribution in several outcome measures, including DASHW4 (*p* = 0.039), NRS Baseline (*p* = 0.007), SFMPQAW4 (*p* = 0.002), SFMPQBW4 (*p* = 0.0003), and PDQW4 (*p* = 0.023). Given these findings, non-parametric tests were employed for further analysis.

Within-group pre- and post-treatment differences were analyzed using the Wilcoxon signed-rank test. Between-group comparisons were performed using the Kruskal–Wallis test, followed by pairwise Mann–Whitney U tests with Bonferroni correction for post hoc analysis. Changes in scores were interpreted based on mean differences and direction of change. Visualizations, including boxplots, were used to illustrate treatment effects across groups.

## 5. Conclusions

There were no statistically significant differences in comparison between the different treatment groups. The constant treatment group showed slightly better average improvements in pain and disability in comparison to the others. In post hoc analysis, a significant difference was observed between groups with constant treatment and without treatment, indicating that the group without treatment had a significantly lower sensory pain score after 4 weeks. Statistically significant improvements in all CRPS patients were observed in pain-related and functional measures, particularly for NRS, DASH, PDQ, and SF-MPQB scores, indicating the potential efficacy of the treatment protocol in reducing pain and improving function.

## Figures and Tables

**Table 1:** Results of complex rehabilitation in all patients with CRPS.

Variable	W-Statistic	*p*-Value	Change	95% CI
DASH	50.0	0.00001	−13.87	[−19.22; −8.52]
LEFS	261.0	0.53246	+1.66	[−2.93; 6.25]
NRS	71.0	0.0001	−1.78	[−2.52; −1.04]
CSIA	93.0	0.00027	−4.19	[−5.78; −2.60]
SFMPQA	186.0	0.02005	10.76	[3.53; 17.99]
SFMPQB1	25.0	0.0	−33.64	[−41.93; −25.35]
PDQ	93.0	0.00079	−3.67	[−6.24; −1.10]

**Table 2:** Changes in parameters in treatment groups (between groups comparison analysis).

Parameters	Group 1 (*n* = 15)		Group 2 (*n* = 6)		Group 3 (*n* = 11)	
	M [CI 95%]	*p*	M [CI 95%]	*p*	M [CI 95%]	*p*
DASH	−14.3 [−23.68, −4.91]	0.0056	−18.71 [−29.92, −7.50]	0.0078	−10.64 [−21.36, 0.08]	0.0515
LEFS	1.14 [−6.43, 8.70]	0.7543	−1.79 [−19.57, 16.00]	0.8065	4.12 [−3.26, 11.50]	0.2447
NRS	−1.69 [−3.07, −0.32]	0.0185	−1.92 [−2.88, −0.95]	0.0037	−1.83 [−3.11, −0.56]	0.0089
CSIA	−1.72 [−5.22, 1.78]	0.3136	−7.00 [−13.01, −0.99]	0.0303	−6.50 [−10.30, −2.70]	0.0031
SFMPQA	8.75 [−3.47, 20.97]	0.1492	5.50 [−13.20, 24.20]	0.4837	16.42 [3.32, 29.52]	0.0186
SFMPQB1	−29.67 [−44.42, −14.91]	0.0006	−33.33 [−63.24, −3.43]	0.0352	−39.75 [−49.81, −29.69]	0.0000
PDQ	−1.56 [−5.53, 2.42]	0.4209	−6.17 [−12.65, 0.32]	0.0583	−5.58 [−10.76, −0.40]	0.0370

**Table 3:** Clinical characteristics of the participants.

Group	Participants (n)	Mean Age (years, M(SD))	Male (n)	Female (n)	DASH	LEFS	NRS	CSIA	SFMPQA	SFMPQB	PDQ
1	15	49.53 (13,2)	3	12	51.29	53.86	5.42	35.06	18.00	62.89	18.33
2	6	61.67 (11,7)	1	5	69.21	47.20	4.83	37.00	21.00	63.67	21.00
3	11	55.10 (14,2)	3	8	55.68	64.94	4.46	32.42	15.00	48.58	18.17

**Table 4:** Pharmacological therapy in Group 1.

Medication Class	Agents Used	Daily Dosage Information	Route
Nonsteroidal Anti-inflammatory Drugs (NSAIDs)	Ketoprofen, Ketonal	100–200 mg	oral
	Ibuprofen	400–800 mg	oral
	Paracetamol	500–1000 mg	oral
Anticonvulsants	Pregabalin	75–150 mg	oral
	Gabapentin	600–1200 mg	oral
Opioid Analgesics	Tramadol	50–100 mg	oral
	Oxycodone	5–10 mg	oral
Combination Therapy	Tramadol + Paracetamol	75/325 mg	oral
	Tramadol + Dexketoprofen	75/25 mg	oral
Serotonin-Norepinephrine Reuptake Inhibitors (SNRIs)	Duloxetine	30–60 mg	oral

## Data Availability

Data is unavailable due to privacy.
